# Who “Believes” in the Gambler’s Fallacy and Why?

**DOI:** 10.1037/xge0000245

**Published:** 2017-01

**Authors:** George D. Farmer, Paul A. Warren, Ulrike Hahn

**Affiliations:** 1 Division of Neuroscience and Experimental Psychology, University of Manchester; 2 Department of Psychological Sciences, Birkbeck, University of London

**Keywords:** Gambler’s Fallacy, randomness, rationality, experience

## Abstract

Humans possess a remarkable ability to discriminate structure from randomness in the environment. However, this ability appears to be systematically biased. This is nowhere more evident than in the Gambler’s Fallacy (GF)—the mistaken belief that observing an increasingly long sequence of “heads” from an unbiased coin makes the occurrence of “tails” on the next trial ever more likely. Although the GF appears to provide evidence of “cognitive bias,” a recent theoretical account (Hahn & Warren, 2009) has suggested the GF might be understandable if constraints on actual experience of random sources (such as attention and short term memory) are taken into account. Here we test this experiential account by exposing participants to 200 outcomes from a genuinely random (*p* = .5) Bernoulli process. All participants saw the same overall sequence; however, we manipulated experience across groups such that the sequence was divided into chunks of length 100, 10, or 5. Both before and after the exposure, participants (a) generated random sequences and (b) judged the randomness of presented sequences. In contrast to other accounts in the literature, the experiential account suggests that this manipulation will lead to systematic differences in postexposure behavior. Our data were strongly in line with this prediction and provide support for a general account of randomness perception in which biases are actually apt reflections of environmental statistics under experiential constraints. This suggests that deeper insight into human cognition may be gained if, instead of dismissing apparent biases as failings, we assume humans are rational under constraints.

Fundamental to the success of the human species is its ability to discern regularities and structure in the world. This allows humans to successfully predict, explain, and manipulate their environment. At the same time, human beings seem to exhibit limitations in discerning patterns that, on occasion systematically lead them astray. Chief among these are misperceptions of randomness such as the Gambler’s Fallacy (GF): the mistaken belief that observing an increasingly long sequence of “heads” from an unbiased coin makes the occurrence of “tails” on the next trial ever more likely. Misperceptions like the GF are of interest because they are consequential, whether in casinos (Croson & Sundali, 2005), racetracks (Terrell, 1998), lottery play (Clotfelter & Cook, 1993), or, possibly most worryingly, in financial markets (Johnson, Tellis, & MacInnis, 2005); see also Rabin (2002). However, the GF also holds theoretical interest far beyond these practical concerns. The mistaken beliefs about randomness embodied in the GF sit somewhat paradoxically with the general success of humans at discriminating (unrewarding) randomness from (potentially valuable) structure in their everyday environment. Consequently, as an error, the GF offers the potential for deep insight into the criteria and cognitive processes by which human beings make judgments about statistical structure—in the same way that visual illusions are informative for understanding perception (see Kahneman & Tversky, 1972). Accordingly, the GF has attracted a long history of research in psychology (for reviews see Bar-Hillel & Wagenaar, 1991; Hahn, 2011; Oskarsson, Van Boven, McClelland, & Hastie, 2009).

There is evidence that problem gamblers believe in the GF, for example from “thinking aloud studies” during gambling, and that this mistaken belief plays a causal role in their gambling behavior (see, e.g., Ladouceur et al., 2001; Toneatto, Blitz-Miller, Calderwood, Dragonetti, & Tsanos, 1997; Toneatto & Ladouceur, 2003). Outside this group, however, the degree to which people endorse the GF is somewhat less clear. This is because—in experimental laboratory studies—participants’ endorsement of the GF has typically only been inferred. Evidence for belief in the GF is *indirect*: it has been inferred from participants’ behavior in random sequence generation tasks. The standard measure on which attribution of the GF has rested is the alternation rate (AR): the probability with which a person alternates between the two outcomes of a “fair” coin toss when generating a “random” sequence has been taken to reflect belief in the GF. While the (long-run) alternation rate of a fair coin is 0.5, many empirical studies have observed that participants tend to “over-alternate,” generating binary sequences with higher ARs (Wagenaar, 1972). This finding is reflected also in sequence judgment tasks: here participants typically perceive sequences with higher ARs to be “more random” Falk and Konold (1997) (though the exact AR regarded as random is affected by context, Matthews, 2013). Overalternation may be taken as evidence of an erroneous belief in the GF (or the “self-correcting” nature of random processes more generally) because it reflects a bias against “runs” or “streaks” of one outcome (e.g., a succession of heads).

This dependence on indirect measures has consequences not only for ascertaining belief in the GF but also for explanation of that belief. It is difficult to distinguish whether overalternations are “accurate reflections of biased notions of randomness, or biased reflections of accurate notions of randomness, or both” (Bar-Hillel & Wagenaar, 1991). This problem is made more acute by the fact that randomness itself is a theoretically vexed notion that holds many counterintuitive surprises (on the many consequences of this for psychological research see, e.g., Ayton, Hunt, & Wright, 1989; Nickerson, 2002).

As testament to the counterintuitive nature of randomness consider the following example. The GF is the clearly erroneous belief that a sequence of heads (H) such as HHH is more likely to be followed by tails (T), than by another H, given an unbiased coin. However, as Hahn and Warren (2009) point out, if we start flipping an unbiased coin, we will (on average) have to wait less long to encounter HHHT than HHHH (Gardner, 1988; Guibas & Odlyzko, 1981; Figure 1A).[Fig-anchor fig1]

More generally the so-called wait times for all 16 binary sequences of length 4 are presented in Figure 1A. Note that there are significant differences between wait times across sequences. These differences in wait time may seem surprising to those encountering the concept for the first time. However, they are a mathematical fact and they have the immediate consequence that the respective probabilities of encountering the sequences HHHT and HHHH are equal only if flipping an unbiased coin exactly four times or infinitely many times. For values in between these two extremes, probabilities will not be the same. Imagine flipping a coin, say, 20 times and checking whether either HHHH or HHHT arise at least once in that series. Given that the wait time for HHHH is longer than that for HHHT, HHHH will also be less likely to occur at all. The probability of not encountering the string HHHH within those 20 tosses of the coin is directly determined by the wait time (Figure 1B). This means that a person who bets $1 on the sequence HHHT occurring at least once in a sequence of 20 coin tosses will, on average, earn more than a person who makes the same bet for HHHH (see Hahn & Warren, 2009, 2010). This bet sounds incredibly similar to erroneous belief in the GF, yet it is mathematically sound, and it provides the basis for a possible explanation of seeming GF-like beliefs.

Inspired by these counterintuitive mathematical results, Hahn and Warren (2009) suggested that constraints on attention and/or short-term memory (STM) may dictate that human experience of random events is actually akin to viewing “local” subsequences in a longer finite “global” stream of events (see Figure 2). Specifically, people will necessarily only ever see finite sequences of outputs from random sources such as unbiased coins, and they will experience that sequence unfolding in time with a limited short term memory that can monitor only a fixed length sequence. Human experience of random events can therefore be thought of as a sliding window containing a local sequence of length *k* moving through a finite global sequence of length *n* > *k*.[Fig-anchor fig2]

The simple experiential model of Figure 2 necessarily results in differences between the number of occurrences of different local subsequences in a longer global sequence (as illustrated in Figure 1). Consequently it may explain seeming “bias”: what appears biased if one is thinking simply about subsequences in isolation, can be seen as reflecting genuine environmental experience once the fact that subsequences arise within longer global streams is taken into account.^1^

Focusing more specifically on the distribution of subsequences HHHH and HHHT, Figure 3 shows that these are very different in global sequences of moderate length. A person will most likely encounter zero occurrences of HHHH in a sequence of length 20. It is only for longer global sequences (Figure 3, Panels e and f) that the frequency of HHHH will tend toward that of HHHT, that is, the two sequences will become alike only with greater experience of the generating source. At the root of all this is the difference in the shape of the underlying distributions for probability of occurrence. While the mean (i.e., the expected frequency) of each distribution is the same for all subsequences, the distributions vary hugely in their skew for global sequences of moderate length. This difference is attenuated only as the global sequence becomes longer (see Figure 3g). In addition the probabilities of nonoccurrence for HHHH versus HHHT become much closer to each other (and to zero) as the length of the global sequence increases. These factors, in turn, suggest that the way people experience sequences of outputs from random sources in everyday life, might explain seeming misperceptions of randomness.^2^

The simple experiential model in Figure 2 and the associated mathematical consequences render the GF comprehensible in that it seems a rather subtle error once placed in the context of the very similar beliefs one could have that, in fact, are accurate, such as the winning bet on HHHT in global sequences of length 20 given above. In addition it is also worth pointing out that the GF is, of course, not a fallacy when sampling without replacement (i.e., repeatedly drawing a ball from an urn with equal numbers of red and blue balls and not replacing the ball after each draw). One explanation for belief in the GF that has been put forward in the literature is that the GF may be based on confusion between sampling with and without replacement (e.g., Ayton et al., 1989). On Hahn and Warren’s simple model of experience, however, sampling with and without replacement are surprisingly similar in experiential terms as shown in Figure 4. Comparing nonoccurrence probabilities of sequences RRRR and RRRB (now representing red and blue balls randomly drawn from an urn in which they are present in equal proportion) shows that the sequence probabilities are virtually unaffected by whether or not sampling is with or without replacement, or the number of balls in the urn initially. In other words, just from limited sequential experience it is almost impossible to distinguish between the two kinds of source. If such a confusion between sampling with and without replacement does indeed exist, the experiential model provides a straightforward explanation why: just from observing random sequences, the difference between the two seems extremely hard to learn.[Fig-anchor fig4]

The Hahn and Warren (2009) account, then, shifts the dominant perspective in randomness perception away from cognitive bias and instead recasts phenomena like GF as unavoidable mathematical consequences of sensitivity to environmental statistics with a constrained window of experience. In this respect it differs fundamentally from previous accounts of the GF and, or, overalternation (Baddeley, 1966; Kahneman & Tversky, 1972; Kareev, 1992; Rapoport & Budescu, 1997, see discussion below). These have attributed the fallacy to biased beliefs that are at odds with statistics such as the “representativeness heuristic” that mistakenly attributes to short sequences the properties of long sequences (Kahneman & Tversky, 1972; Rapoport & Budescu, 1997). Or they have attributed overalternations in sequence generation to resource constraints distorting an underlying, unbiased conception of randomness itself (e.g., Kareev, 1992). The present account takes up the idea of resource constraints, but asks how these constraints shape people’s actual experience of random sequences. In so doing, it identifies ways in which seemingly biased beliefs about randomness are, in fact, correct and represent reflections of (experienced) environmental statistics.

This, finally, also has implications for the assessment of explicit belief in the GF. Shorter sequences have ARs that lie above the long run average (see also Kareev, 1992). Consequently overalternation may simply be a reflection of the statistics of random sequences as experienced. However, there is no reason why this should necessarily be coupled with explicit belief in the GF. Therefore, it is essential to probe whether or not people endorse the GF with other measures than just AR; in particular it would seem appropriate to supplement indirect, implicit measures such as AR, with direct, explicit probes concerning people’s beliefs.

Taken together these considerations suggest that if the prevalence and causes of the GF are to be understood then there is a clear need for experimental studies that: (a) probe the role of experience in the GF; (b) test the experiential model of Hahn and Warren (2009); and (c) consider the extent to which direct and indirect measures of belief in the GF are in agreement. We next outline an experimental paradigm to address these questions.

## An Experimental Test

Crucially under the Hahn and Warren (2009) account, not only should experience of the output of random sources be expected to modify perception of randomness but also global sequence length (parameter *n* in Figure 2) should matter. This critical role of global sequence length provides a basis for an empirical test of a general role for experience in the GF and of the specific experiential model of Hahn and Warren (2009), thereby addressing points (a) and (b) above.

**Figure 1 fig1:**
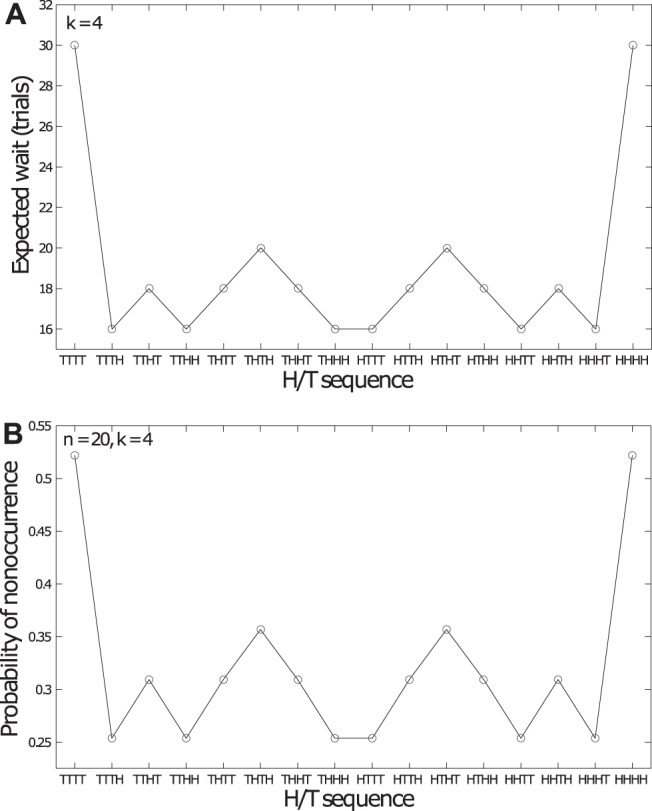
(A) Expected wait time for subsequences of length 4 and (B). the associated probability of not encountering each sequence in a longer “global” sequence of length 20. The wait time statistic directly determines the nonoccurrence probability—the longer the wait time, the higher the probability of nonoccurrence in a longer, finite string. Reproduced from Hahn and Warren (2009) p. 456.

To see the effect of global sequence length consider Figures 3a to f, showing distributions of occurrence frequencies of both HHHH and HHHT for three values of *n* (20, 50, and 200). Note that for each global sequence length the mean number of occurrences of the two subsequences is the same, however, the frequency distributions are markedly different. It is precisely this difference in distributions that drives the difference in wait times and nonoccurrence probabilities seen in Figure 1. The primary difference between HHHH and HHHT occurrence distributions is in the skew. Both distributions show positive skew at each global sequence length; however, this is more pronounced in the case of HHHH. The increased skew for HHHH reflects that fact that although frequency is bounded below by 0 for both subsequences the spread in the HHHH distribution is greater. Consequently, for a global sequence of any given length it is more likely that there will be very few occurrences of HHHH than very few occurrences of HHHT. Figure 3g summarizes the relationship between skew and global sequence length and suggests that this tendency for differences between HHHH and HHHT distributions decreases with global sequence length. In summary, Figure 3 suggests that exposure to longer global sequences should diminish discrepancies in the frequencies with which HHHH and HHHT are encountered.

A manipulation of global sequence length could thus be at the heart of an experimental test of the experiential model: experience of longer global sequences should reduce differences in perceived randomness of HHHH and HHHT. Global sequence length, however, is also naturally correlated with the overall amount of experience: presenting one participant with a global sequence of length 20 and another participant with a sequence of length 200 also means the latter receives 10 times as much experience of a random source. A better experimental manipulation would thus seek to manipulate global sequence length while keeping constant the overall amount of exposure to a random generating source.

This can be achieved by providing several shorter global sequences so as to match the total amount of experience: a participant who sees 10 global sequences of length 20 will see the same number of coin tosses as one who sees 1 global sequence of length 200. Yet, as Figure 3 shows, the distribution of HHHH and HHHT will be different under these two presentation conditions (e.g., compare Figures 3a and 3b to Figures 3e and 3f).

For even greater experimental control, one can literally take the same total exposure as represented by a particular finite sequence of coin tosses and divide it up into global sequences of different length to generate differences across sequences for the experiential model: Specifically, in a sequence of length 200 a person with a sliding window of length 4 could observe a maximum of 197 runs of HHHH. However, when that same sequence is divided into 20 global sequences of length 10, the maximum number of HHHH runs that can be observed is only 140. Figure 5 shows this relationship for the same total experience of 200 coin tosses discretized into “global sequence” chunks of varying size. The way the same overall experience is divided up into global sequences determines an upper bound on the number of runs a person could ever observe as the attentional window moves through these global sequences.[Fig-anchor fig5]

These considerations provide a simple test whereby the same total exposure is presented to participants in subtly different ways: Specifically, the same 200 coin tosses might be presented as 2 global sequences of length 100 or, alternatively, as 10 smaller global sequences of length 20. The difference in experience for the observer is rather subtle—exactly the same set of outcomes is observed but in blocks of different lengths with gaps in between. However, under the Hahn and Warren (2009) account this should lead to different behavior in a randomness perception task. Specifically, increasing the length of global sequences should lead to increased tendency to produce runs of identical outcomes (leading to reduced AR) in a generation task or to judge a run of identical outcomes as random in a judgment task.

**Figure 2 fig2:**
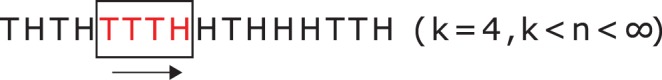
The sliding window account from Hahn and Warren (2009) p. 455. People’s experience of random sequences is theorized to be a local, sliding window through a global finite sequence. See the online article for the color version of this figure.

Our study tests whether the subtle manipulation of global sequence length suggested above leads to changes in both the judgment and generation of random sequences. We presented the same length-200 sequence to all participants but manipulated whether they saw it in 40 chunks of length 5, 20 chunks of length 10 or 2 chunks of length 100. Chunk size is therefore analogous to the global sequence length *n* in Figure 2, and we expect that exposure to different *n* will be reflected in the extent to which people produce data consistent with GF measures. In a random sequence generation task we measured the AR and the GF ratio (i.e., the ratio of HHHH to HHHT) for occurrences of subsequences of length 4.

**Figure 3:**
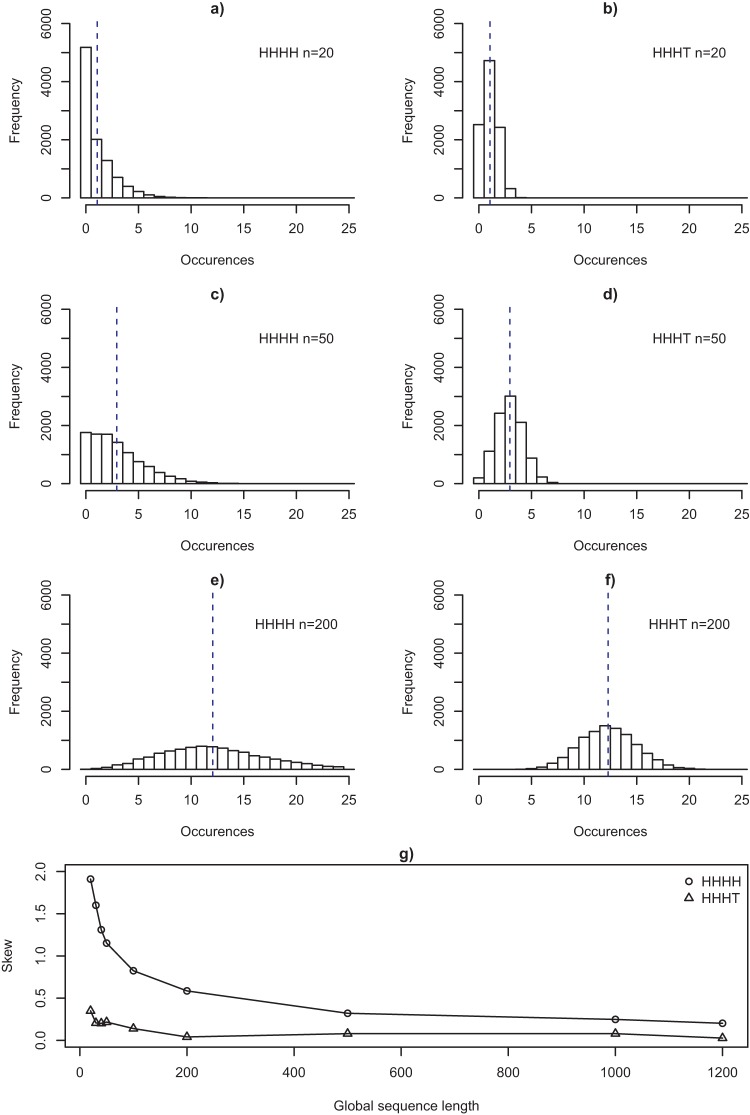
(a–f) The number of occurrences of a local subsequence within a global sequence of length *n* = 20 (Panels a and b), *n* = 50 (Panels c and d), and *n* = 200 (Panels e and f) for subsequences HHHH and HHHT. H = heads; T = tails. Vertical dashed lines indicate the mean number of occurrences. (Panel g) Skew of the sampling distributions of HHHH and HHHT for global sequence lengths *n* = 20 to *n* = 1,200. Panel g from Hahn (2014) p. 234.

Based on Figure 3 we expect that the commonly observed underemphasis of runs (H . . . H) will diminish as chunk size increases and behavior will become more in line with normative properties of binomial sequences. Accordingly, as chunk size increases, AR should decrease, moving closer to the normative value of 0.5, and the GF ratio should increase moving toward the normative value of 1. Furthermore, in a randomness judgment task we used Falk and Konold’s (1997) method to assess which AR was perceived as most random. We predict that as chunk size increases the AR judged as most random will again decrease and move closer to 0.5.

In addition to focusing on the sequences HHHH and HHHT, which are particularly relevant for the GF, we also consider the other 14 local subsequences of length 4, assessing whether Hahn and Warren (2009) predicts their prevalence in the generation task. Finally, to address point (c) above we assessed the relationship between explicit beliefs in the GF and the indirect measures (such as AR) commonly assumed to be equivalent to belief in the GF. To do this we developed a short questionnaire that probed participants directly on their explicit beliefs in GF.

## Method

### Participants

One-hundred and eighteen people volunteered to take part from the University of Manchester student and staff population. Participants gave informed consent, and received course credit or £7.50 as reimbursement for their time. Participants’ mean age was 21, *SD* = 4.8, 77% of participants were female. There were no exclusion criteria.

### Materials

Participants were seated in front of a 19” monitor at a 1,280 × 1,024 resolution. Participants made responses using a standard Windows keyboard.

### Design

**Figure 5 fig5:**
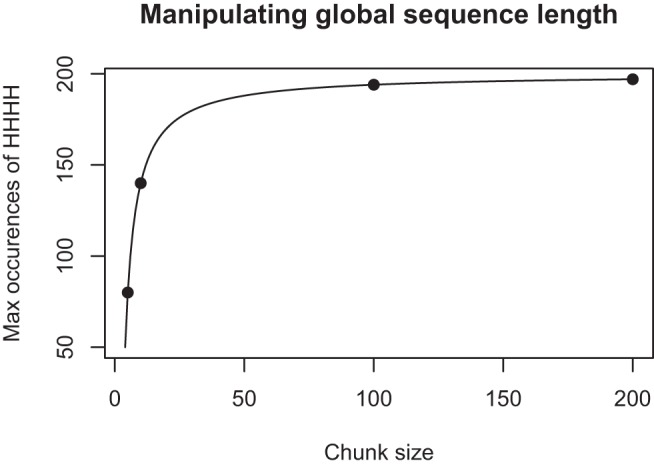
Maximum number of subsequences HHHH that could be encountered when the same length-200 sequence is chunked into different sizes. Points show 40 × 5, 20 × 10, 2 × 100, and 1 × 200.

Within subjects, participants generated and judged a sequence both before and after being exposed to sequences generated by a .5 Bernoulli process. Between subjects we manipulated the nature of the exposure by chunking it into different sized blocks. There were three levels of this chunk size IV (100, 10, and 5). Overall this resulted in a two within (exposure: pre, post) × three between (chunk size: 100, 10, and 5) design. Forty participants were in the 20 × 10 condition, 39 in the 2 × 100 and 39 in the 40 × 5 condition. All participants were presented with the same 200 coin tosses in succession. This series was generated by a Bernoulli process, but was checked to ensure that it had a representative AR of approximately .5. The nature of the exposure differed only in terms of the size of chunk the series was divided into, 2 × 100, 20 × 10 or 40 × 5. The gap between chunks was very short, typically around 1 s. Chunking in this manner gave rise to global sequences (see Figure 5) of varying length while still controlling for the overall amount and content of experience.

### Procedure

The experiment consisted of both generation and judgment of random sequences. These were repeated before and after an observation phase in which participants saw output from a genuine random source. At the end of the experiment participants completed a questionnaire designed to elicit their beliefs about the gambler’s fallacy. This included asking people how they would bet after a sequence of five heads in a row, and whether after five heads: heads was most likely, tails was most likely, or both were equally likely.

#### Generation task

Participants were asked to generate a series of coin tosses by pressing 1 for heads and 0 for tails on the computer keyboard. They were instructed to produce a sequence that they thought would be representative of flipping a fair coin. Participants were encouraged to type at a speed of roughly 1 press per second, and they could see an ‘H’ or ‘T’ appear on the screen. Each display of H or T replaced the previous display so participants could not see the history of their sequence. Participants were instructed to generate a sequence of length 200 in 2 blocks of 100. In between each block the screen would display the message “sequence 2 of 2:” and required the participant to acknowledge it by pressing ‘c’ to continue.

#### Judgment

The judgment task was adapted from Falk and Konold (1997). Ten sequences of heads and tails were used. Each sequence was of length 21 but varied in AR from 0.1 to 1. The participants’ task was to rate each sequence according to how likely it was to have been produced by a fair coin. Participants were required to start by giving scores of 0 and 10 to the least and most likely sequences, respectively. They were then free to score the other sequences relative to these and they could use the same score more than once. The pre- and postobservation judgment tasks used the same ARs but different sequences.

#### Observation

Participants were told: “Some people find it difficult to generate random sequences. You are now going to see a genuine random sequence that would be produced by a fair coin. You should pay attention to the sequence, and then you will be asked to generate a new sequence” An ‘H’ or ‘T’ was presented with a SOA of 700 ms. Each presentation replaced the previous one so that the history of the sequence was not visible. All participants experienced the same sequence except that in different conditions the number of breaks changed (1, 19, or 39). A break consisted simply of the participant pressing the letter ‘c’ on the keyboard to continue, this typically took around 1 s.

### Analyses

For all analyses we compare the sequences that participants generated before and after they were exposed to the genuine random sequence. In this way we can test the impact of the exposure on the different metrics associated with generation and judgment of random sequences.

We also gave participants a questionnaire designed to probe their beliefs around the GF. We classify participants who stated that a tails is more likely after a run of heads as “believers” in the GF. We compare the metrics or AR and the ratio HHHH:HHHT between the believers and nonbelievers. For these analyses we use the pre-exposure data of our participants.

## Results

### Generation

**Figure 6 fig6:**
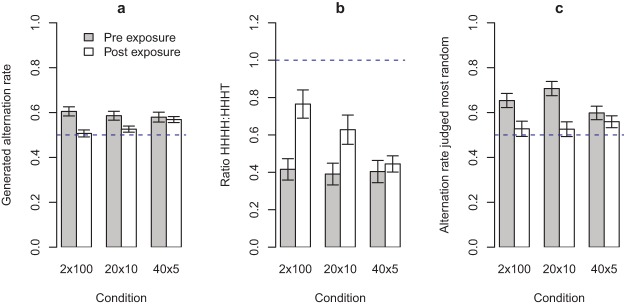
(a) Alternation rate pre and postexposure for each of the chunking conditions. The largest reduction in alternation rate was in the 2 × 100 group and the smallest in the 40 × 5 group. (b) Ratio of runs of length 4 to runs of length 3 plus an alternation for sequences produced in each condition (this includes HHHH:HHHT and TTTT:TTTH). The 2 × 100 condition saw the largest increase in ratio, while the 40 × 5 condition saw the smallest increase. (c) Alternation rate judged most likely to be produced by a fair coin in each condition. In all panels the horizontal dashed line shows the theoretical value for a fair coin. Error bars represent ±1 *SE*.

Participants’ raw data consisted of the self-generated sequences of length 200, produced in two blocks of 100. For each participant, we measured their AR over the entire 200 bit sequence, that is, we calculated the number of switches between H and T as a proportion of a maximum 198. Analysis of the AR in generated sequences before exposure showed that participants typically overalternated at around 0.6, consistent with the literature (reviewed in Falk & Konold, 1997). Figure 6a shows the change in AR from the pre-exposure to postexposure generation. In line with our predictions, the AR decreased most in the 2 × 100 condition and least in the 40 × 5 condition. A mixed analysis of variance (ANOVA)^3^ revealed a significant effect of exposure (pre, post) *F*(1, 115) = 18.51, *p* < .001, η_G_^2^ = .06, and a significant interaction (exposure × chunk size) *F*(2, 115) = 3.72, *p* = .027, η_G_^2^ = .03. There was a nonsignificant main effect of chunk size *F*(2, 115) = .59, *p* = .56. Bonferroni corrected *t* tests (α = .016) for each of the three conditions revealed that the effect of exposure was significant in the 2 × 100 (*p* < .01) and 20 × 10 (*p* = .01) conditions, but not in the 40 × 5 condition (*p* = .63).[Fig-anchor fig6]

Using the same data we also checked for a different measure indicative of the GF: we measured the ratio of sequences containing a run of length 4 to those containing a run of length 3 with an alternation that is, HHHH:HHHT and TTTT:TTTH. This analysis revealed that pre-exposure participants typically produced twice as many HHHT sequences. Figure 6b shows the ratios pre-exposure and postexposure (three participants were excluded from this analysis as a ratio could not be calculated because of never generating long-enough runs). In line with our predictions, in the 2 × 100 condition the ratio increased by 0.35 to 0.77, while in the 40 × 5 condition the ratio increased by just .05 to 0.45. A mixed ANOVA revealed a significant effect of exposure (pre, post) *F*(1, 112) = 20.27, *p* < .001 η_G_^2^ = .07, and a significant interaction (exposure × chunk size) *F*(2, 112) = 3.78, *p* = .026, η_G_^2^ = .03. There was a nonsignificant main effect of chunk size *F*(2, 112) = 2.91, *p* = .058. Bonferroni corrected *t* tests (α = .016) for each of the three conditions revealed that the effect of exposure was significant in the 2 × 100 (*p* < .01) and 20 × 10 (*p* = .01) conditions, but not in the 40 × 5 condition (*p* = .51).

**Figure 7 fig7:**
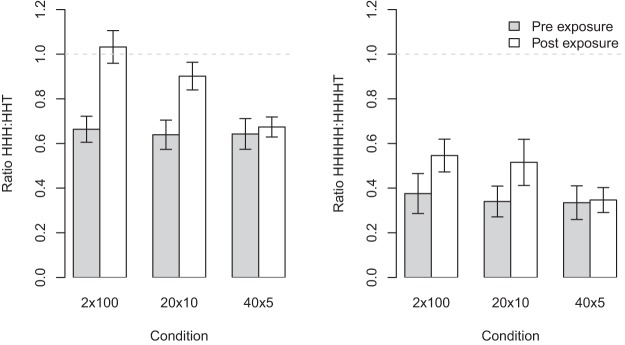
Left panel: Change in the ratio of runs of length three to runs of length two plus an alternation for sequences produced in each condition (this includes HHH:HHT and TTT:TTH). Right Panel: The same analysis for runs of length five and runs of length 4 plus an alternation. In both panels the 2 × 100 condition shows the largest increase in ratio, while the 40 × 5 conditions shows the smallest increase. The horizontal dashed line shows the theoretical value for a fair coin. Error bars represent ±1 *SE*.

**Figure 8 fig8:**
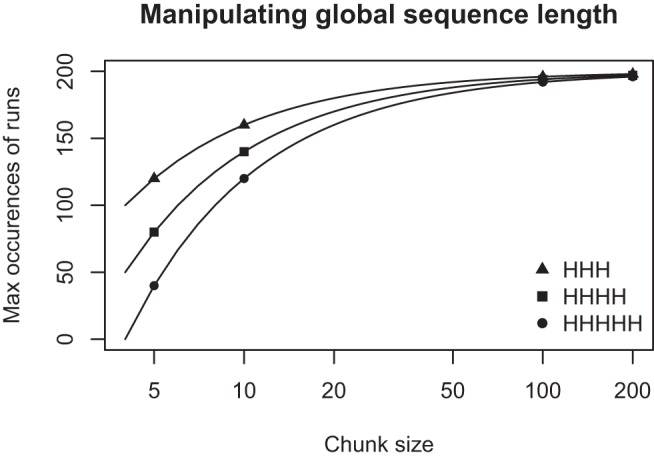
The maximum number of times a run of heads (or tails) can occur given different discretization of a 200 long sequence. The longer the run length the fewer occurrences are expected. Data points represent discretization at chunk sizes 5, 10, 100, and 200. Note the *x*-axis is log scale.

Figure 7 shows the effect of exposure on participants’ production of runs of length three and five. In both cases the pattern is the same as for runs of length four. For the analysis at length five there is a loss of power because participants rarely produced runs of this length. Figure 8 shows that the maximum number of occurrences of runs decreases as a sequence of length 200 is discretized into smaller chunks. The same pattern is observed for different lengths of run, but is more pronounced for longer runs.[Fig-anchor fig7][Fig-anchor fig8]

### Judgment

**Figure 9 fig9:**
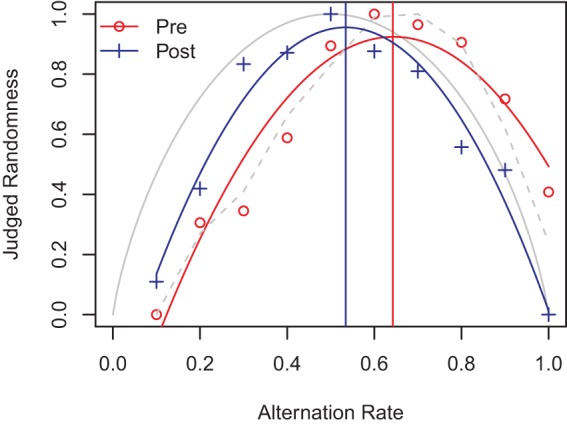
A quadratic function fitted to pre and post judgments at the group level. The peak of the postexposure judgments has shifted toward the theoretical value of 0.5. The vertical lines show the peak of the fit. The gray dashed line shows the original data collected by Falk and Konold (1997) p. 314, a close match to our pre-exposure data. Solid gray line shows second-order approximate entropy (Beltrami, 1999) of the alternation rate. See the online article for the color version of this figure.

Participants’ raw data were relative rankings of how likely they perceived each of the 10 test sequences to have been produced by a fair coin. When AR is plotted against the judged randomness the data follows an inverted ‘U’ shape Falk and Konold (1997) because sequences with either high or low ARs look nonrandom. For each participant we fit a quadratic function to their judgment data and recovered the peak of the fitted curve for their pre-exposure and postexposure data. Thus, for each participant we obtained the AR they judged most random before and after exposure (i.e., the peak of the fitted quadratic). For the purpose of illustration, Figure 9 shows the aggregate level fits (i.e., over data from all observers) for randomness judgments in the 20 × 10 condition.[Fig-anchor fig9]

Before any exposure people typically rated the 0.6 alternation sequence as the most likely to have been produced by a fair coin. This is consistent with other results in the literature (e.g., Falk & Konold, 1997; Zhao, Hahn, & Osherson, 2014). Figure 6c shows how the fitted peak position changed from pre-exposure to postexposure in each condition (averaged over observers). The 2 × 100 and 20 × 10 conditions exhibited similar shifts in judgment toward 0.5 as the AR most likely to have been produced by a fair coin. The 40 × 5 condition revealed a smaller shift. A mixed ANOVA revealed a significant effect of exposure (pre, post) *F*(1, 115) = 32.38, *p* < .001, η_G_^2^ = .08, and a significant interaction (exposure × chunk size) *F*(2, 115) = 4.17, *p* = .018, η_G_^2^ = .02. There was a nonsignificant main effect of chunk size *F*(2, 115) = .56, *p* = .57. Bonferroni corrected *t* tests (α = .016) for each of the three conditions revealed that the effect of exposure was significant in the 2 × 100 (*p* < .01) and 20 × 10 (*p* < .01) conditions, but not in the 40 × 5 condition (*p* = .21.). This matches the findings of the generation task.

### Explicit GF Beliefs and Indirect Measures

Participants were asked how they would bet on a fair coin after a sequence of five heads. Eighty per cent responded that they would bet tails. Participants were also asked which outcome was most likely following five heads. Of those that bet tails to the initial question, 75% answered that both outcomes were equally likely, while the remainder responded that tails was more likely. All participants were asked whether they found it counterintuitive that a run of heads did not mean an increased probability of a tails on the next flip. Eighty-seven per cent of participants indicated that they did find it counterintuitive.

**Figure 10 fig10:**
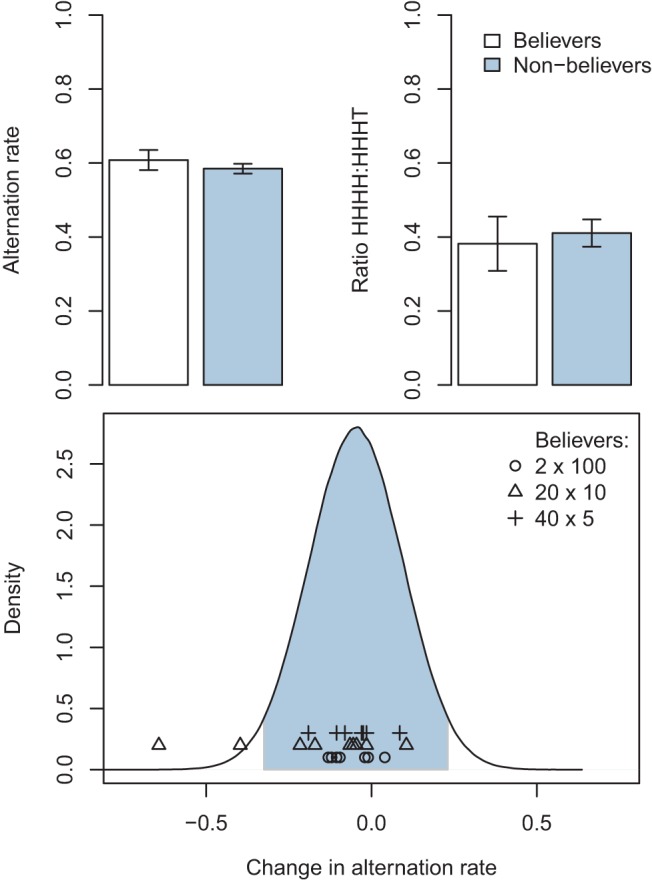
Top panels: Analysis of the implicit measures of the Gambler’s Fallacy (alternation rate and ratio HHHH:HHHT) in participants who explicitly either believed in the fallacy or not. No difference was found on the implicit measures between believers (*n* = 27) and nonbelievers (*n* = 91). These analyses were conducted on participants’ pre-exposure generated data. Bottom panel: Distribution of change in alternation rate from pre- to postexposure for the nonbelievers. The believers are superimposed as data points. The shaded area represents the central 95% of the distribution. See the online article for the color version of this figure.

We label the participants that stated a tails outcome was more likely after a run of heads as believers in the GF. We then probed potential differences between believers and “nonbelievers,” see Figure 10. First, we sought to examine the link between participants’ explicitly stated beliefs and the implicit measures of AR and the ratio HHHH:HHHT. An examination of the AR among believers and nonbelievers in their pre-exposure sequences shows that there was no significant difference between the two groups. Likewise, an examination of the ratio HHHH:HHHT showed no difference between believers and nonbelievers. To support the conclusion that there was no difference between the groups on these measures we conducted a Bayes Factor *t* test (Rouder, Speckman, Sun, Morey, & Iverson, 2009). For the AR test of believers versus nonbelievers: Bayes factor = 3.38, *t* = 0.77, *p* = .444 indicating that the null hypothesis (no difference) was around three and a half times more likely than the alternative hypothesis given the data. For the ratio HHHH:HHHT test of believers versus nonbelievers: Bayes factor = 4.15, *t* = −0.35, *p* = .729 again supporting the conclusion that there was no difference between the groups. In short, individual differences with respect to AR (see also Budescu, 1987) were unrelated to explicit beliefs about the GF.[Fig-anchor fig10]

### The Experiential Account and Rival Theories of Randomness Perception

The results thus far indicate clearly that AR is unrelated to explicit GF belief. Furthermore, it is modified by experience, and global sequence length moderates the impact of experience. The decoupling between explicit belief and AR suggests that AR is not a suitable measure for belief in the GF. Nevertheless, overalternation remains a feature of human randomness perception that needs to be explained. The moderating influence of global sequence length is a unique prediction of the Hahn and Warren (2009) experiential model. Consequently, that result boosts the experiential model over other, rival accounts. However, there are also other facets of the data that are relevant to adjudicating between rival theories, not just of the GF but of randomness perception more generally. We next highlight these aspects.

The majority of past studies of human randomness perception have involved sequence generation tasks (e.g., Kareev, 1992; Nickerson & Butler, 2009; Rapoport & Budescu, 1997; Wagenaar, 1972). A smaller number of studies has used sequence judgment tasks (e.g., Kahneman & Tversky, 1972). Even fewer are studies such as ours that have examined sequence generation and judgment together. This matters because empirically adequate accounts of randomness perception have to be able to explain both generation and judgment. Yet, many theoretical treatments have focused exclusively on one or the other.

As outlined in the introduction, the key theoretical tensions between different accounts have centered on the contrast between “accurate reflections of biased notions of randomness, or biased reflections of accurate notions of randomness” (Bar-Hillel & Wagenaar, 1991). An example of the latter is Kareev’s (1992) account that attributes overalternation in sequence generation tasks to STM limitations.^4^ In effect, participants have an adequate notion of randomness, but overalternations arise because they can plan only in relatively short sequences, and short sequences necessarily have ARs that lie above the long run average. As evidence for this, Kareev (1992) shows correlations between AR and measures of STM. Kareev’s insight on the link between sequence length and AR is profound and, as seen above, figures also in the experiential model. However, Kareev’s use of the concept does not go far enough. There is nothing on Kareev’s account that would predict matching findings regarding AR for both generation and judgment, given that a sequence presented for judgment can be scanned and rescanned at will, and does not require planning in the way that sequence generation does. Yet, the data on AR match closely across judgment and generation.

**Figure 11 fig11:**
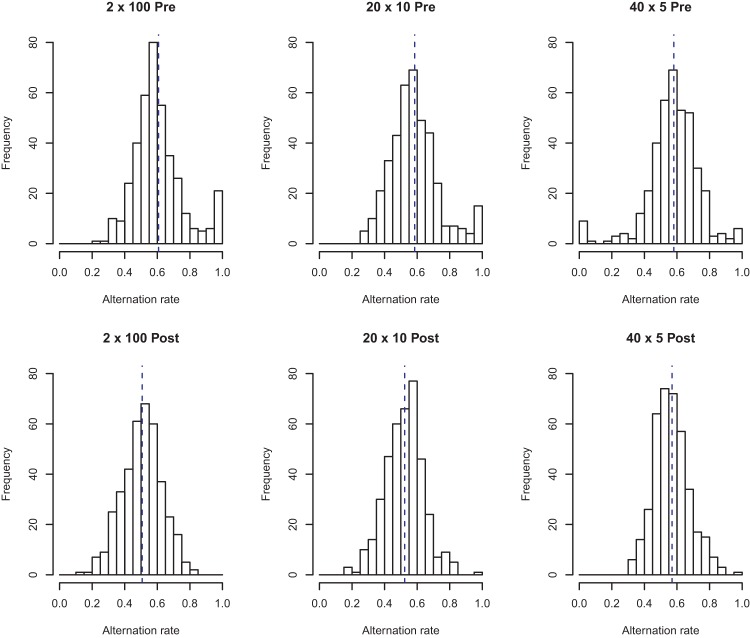
The alternation rate of participants’ generated sequences. Each participant’s pair of 200-long generated sequences were split into 10 sequences of length 20. These were aggregated within each condition to produce the above histograms. The vertical dashed lines indicate the mean of the distribution. In the 2 × 100 and 20 × 10 conditions, the mean moves closer to the theoretical value of 0.5 postexposure.

To illustrate this point, we divided the generation data from our participants into sequences of length 20, so as to match the sequences seen in the judgment task. We then plotted the resultant data in histograms representing the number of length 20 sequences generated with each AR, shown in Figure 11 below. The shape and moments of the resultant distributions can be compared with the judgment data shown in Figure 9 above, and reveal a close correspondence across judgment and generation.[Fig-anchor fig11]

This correspondence argues against the notion that overalternations simply reflect information processing limitations that are preventing people from expressing their true, underlying conception of randomness (cf. also Baddeley, 1966). Instead, it suggests that the underlying conception of randomness itself is influenced by STM in the way the experiential model assumes.

It is only on the experiential account, furthermore, that the effect of exposure can be explained. There is no reason to assume that participants’ STM capacity is affected by the 200 bit total exposure to a random source which they receive in our experiments. Therefore, an STM-based planning limitation necessarily leaves the shift in AR from pre- to postexposure seen in our participants in both generation and judgment (Figures 9 and 11) data entirely unexplained.

Finally, Figures 9 and 11 are also informative with respect to another account of human randomness perception. Whereas most psychological theories have focused on “randomness” as defined by the nature of the underlying generating source, Falk and Konold’s (1997) account views randomness as a property of sequences themselves, in line with theoretical accounts of randomness based on algorithmic complexity (see Beltrami, 1999 for an introduction to the latter). From the perspective of algorithmic complexity, sequences are random to the extent that they are incompressible, that is, they cannot be given descriptions that are shorter than simply listing the sequence itself. Short descriptions can capitalize on inherent structure; randomness—as the opposite of structure- does not admit of short descriptions because there are no regularities to summarize. Falk and Konold (1997) draw on such notions to suggest a psychological notion of randomness based on the notion that the more “regular” a sequence, the easier it is to encode either in verbal description or memory. Specifically, people use memorability of sequences as a proxy for randomness in their judgments of randomness. Following Falk and Konold (1997) Figure 9 above plots AR against approximate entropy, a sequence based measure of randomness. Falk and Konold (1997) concluded that the second-order approximate entropy (a measure of minimum description length, see Beltrami, 1999) provided a reasonably good (although biased) description of their randomness judgment data. This entropy metric quantifies the average information contained in a bit, given knowledge of the preceding bit. Low and high entropy then reflect either compressible or incompressible sequences, respectively. Visual inspection suggests a reasonable enough fit between the sequences and their approximate entropy in our data also (see solid gray line in Figure 9). Furthermore, the role of exposure would seem to be to bring judgments into *greater* alignment with approximate entropy, at least at first glance (although the effects of our global sequence length manipulation are, of course, left unexplained).

**Figure 12 fig12:**
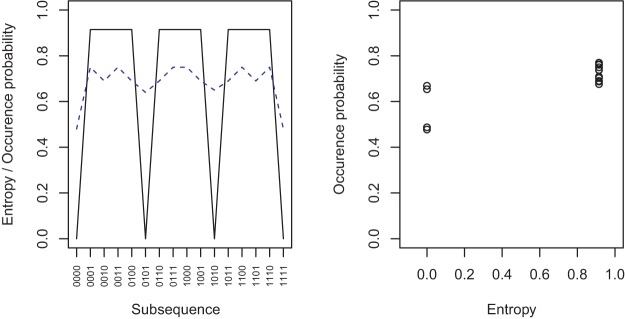
In the left panel, the solid black line shows the entropy based on the alternation rate of each possible subsequence of length 4. The dashed blue line shows the occurrence probability under the sliding window account (Hahn & Warren, 2009). The right panel shows the relationship between the two measures. The occurrence probability values have jitter added since the symmetry (see left panel) means the points actually overlap perfectly.

To compare further the comparison between entropy and the experiential model, we examined the entropy of the length 4 sequences that make up the sliding window on the experiential account. The entropy measure is, to some extent, correlated with the predictions of the Hahn and Warren experiential model, but there are noticeable differences. These are illustrated in Figure 12 that plots the differences between all possible sequences of length 4 in terms of their wait time based differences (blue dashed line, representing theoretical occurrence rate for *k* = 4, *n* = 20; see also Figure 1 a above) and their respective differences in terms of approximate entropy (black line). In particular, entropy makes fewer distinctions between the sequences than does wait time/occurrence rate. This suggests the possibility of a comparison between entropy (and thus Falk and Konold’s algorithmic complexity based account) and the experiential model.[Fig-anchor fig12]

**Figure 13 fig13:**
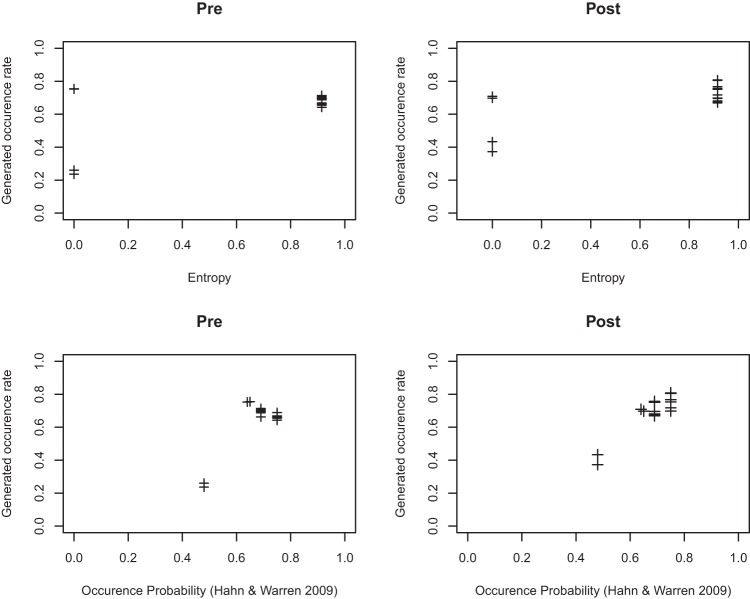
Each data point is 1 of 16 possible subsequences of length 4. The rate with which participants generate each of these sequences is poorly predicted by entropy, but well predicted by the Hahn and Warren (2009) account. The postexposure generated occurrence rate fit with Hahn and Warren is *r* = .92 versus 0.67 for Entropy.

We used the 20-bit sequences of Figure 11 and counted, across the set of sequences, the occurrence of each of the 16 possible subsequences of length 4 within a sliding window moving through the global length 20 sequence. Figure 13 shows the correlations obtained (both pre- and postexposure) between the observed occurrence rates and the theoretical predictions of the experiential model alongside the corresponding correlations between observed occurrence rates and entropy. The pre occurrence rate correlation with entropy (*r* = .52) was lower than the postexposure correlation (*r* = .67). However, in both pre- and postexposure the correlation with Hahn and Warren (HW) was higher (pre *r* = .82, post *r* = .92). We compared the postexposure correlations using Lee and Preacher’s (2013) method. The HW postexperience correlation was significantly greater than the entropy postexperience correlation (*z* = 3.30, *p* < .01). The experiential model clearly provides a better fit.[Fig-anchor fig13]

## Discussion

A very limited amount of experience (exposure to 200 outcomes) materially changed perception of random sequences. Both the sequences generated and those judged to be most random were significantly different from those obtained pre-exposure. Furthermore, participants’ generation and judgment tendencies typically taken as indicative of the GF were significantly reduced postexposure. This is notable, first of all, because all participants did was passively observe 200 flips of a coin; there have been some demonstrations of learning with respect to randomness perception, but these have all involved response specific feedback (e.g., Edwards, 1961; Neuringer, 1986; Rapoport & Budescu, 1992), not mere observation of a very limited sequence of outputs. Moreover, a subtle change in the way the experience was delivered was consequential. Differences in learning rates across the sequence chunking conditions indicate that it is necessary to take the actual nature of experience into account in understanding human randomness perception, as assumed by the account.

For the conditions in which people experienced chunks of length 10 or 100, both sequence generation and sequence judgment-based metrics became more closely aligned with the normative properties of binomial sequences, while there was no significant change for sequences of length five. This was the case even though all participants saw exactly the same series of coin tosses overall. This lends support to the notion that memory capacity and, or, attention gives rise to a sliding window moving through a global, temporally unfolding sequence experience and that this plays a causal role in people’s perceptions of randomness.

Why then was there no change in the 40 × 5 condition? The fact that there *was* change in the 20 × 10 condition indicates clearly that the lack of change cannot simply be based on the fact that the test generation conditions (produce 2 × 100) fail to match the training environment (40 × 5). This is further confirmed by the parallel results across generation and judgment, because the judgment task involves sequences of yet another length (not experienced in training in any of the conditions). This suggests as an explanation that the lack of change in the 40 × 5 condition stems from the fact that this condition might be close to their day-to-day experience of binomial sequences such as coin flips. As a consequence, participants in this condition effectively experience little that is “new.”

Finally, not only was GF-like behavior readily modified by participants’ limited exposure to a random sequence, but the questionnaire data revealed that most participants did not explicitly endorse the gambler’s fallacy. While a large majority stated they would bet on tails following a run of heads, the majority of those also report that there is no difference in likelihood between heads and tails. The fact that people further admit to finding this counterintuitive can be explained as a contrast between what they know to be a declarative fact, and knowledge they have gained from experience. This sits well with notion that seeming biases in randomness perception such as the GF reflect perceived environmental statistics, and makes clear that the inferential leap from seeming bias on implicit measures to supposedly mistaken conceptions of randomness are deeply problematic. Our data suggest that the ARs of generated sequences do not allow us to distinguish between people who do and do not explicitly believe in the GF. This, in turn, suggests that claims of human “irrationality” in this context may have been considerably overstated. It also suggests that very specific explanations will be required to understand the minority who do explicitly endorse the fallacy (a group likely to include problem gamblers, see Källmén, Andersson, & Andren, 2008; Ladouceur et al., 2001; Toneatto, Blitz-Miller, Calderwood, Dragonetti, & Tsanos, 1997; Toneatto & Ladouceur, 2003).

Taken together, our results also pose problems for other accounts of the GF in the literature. Possibly the most well-known of these is Kahneman and Tversky’s (1972) explanation in terms of the so-called “representativeness heuristic.” It assumes that people fail to appreciate how much the properties of short sequences differ from the statistical properties of very long sequences of outputs from a random source.

As a result, they wrongly attribute long-run properties (as captured by the Law of Large Numbers), such as likely equal numbers of heads and tails, to short sequences and perceive as “random” those sequences that seem most “representative.” Though the representativeness heuristic has been criticized as vague in other judgment contexts (see, e.g., Gigerenzer, 2009) it has been formalized in the context of randomness perception (e.g., Rabin, 2002; Rapoport & Budescu, 1997). Somewhat ironically, Hahn and Warren’s (2009) experiential account and the present data suggest that the differences between properties of short sequences and the long run are indeed key to understanding human perceptions of randomness, but not because people’s beliefs about shorter sequences are mistaken, but rather because the properties of shorter sequences determine people’s actual experience. It is compatible with this latter perspective that participants’ sequence judgment and generation was modified by experience, and modified only by presentation in the form of longer global sequences. Under the representativeness heuristic one would expect the long global sequences to be most similar to the participants’ expectations of random sequences. However it was experience with the long global sequences that produced the biggest change in behavior.

Relatedly, the present data also rule out accounts of overalternation based on the idea that these are generated by short-term limitations constraining sequence generation (e.g., Baddeley, 1966; Kareev, 1992).^5^ Precisely because the AR of short sequences lies above the long run average, overalternations would be expected from an unbiased agent who was limited by STM to effectively generate a long sequence of “random outputs” by stringing together shorter sequences that can be held in working memory (for one way of modeling such a process see Rapoport & Budescu, 1997). The fact that participants in the 2 × 100 experience condition subsequently dropped their AR to the long run average of .5 suggests that it is not a performance limitation that is responsible for overalternations in generation tasks. And it is only the experiential account that explains the fact that exactly the same thing happens in those participants’ judgments of random sequences.

An alternative approach to understanding human randomness perception stems from theoretical accounts of randomness based on compression and algorithmic complexity (see, e.g., Beltrami, 1999 for an introduction). These seek to characterize randomness not in terms of the sequence generating source, but in terms of the resultant sequences themselves. Specifically, sequences are random to the extent that they are incompressible, that is, they cannot be given shorter descriptions than enumerating the sequence itself by exploiting sequence structure. Falk and Konold (1997) suggest that people judge a sequence to be less random the more regular and hence easier it is to encode either in verbal description or memory. In other words, memorability of sequences provides a proxy for randomness. This means that long streaks or runs, like other regularities, are viewed as indicative of nonrandomness, which in turn provides an (indirect) explanation of overalternations. While our participants’ initial judgment data matches Falk and Konold’s (1997) data for those same sequences, the postexperience shift is left entirely unexplained.

Finally, the present data also caution against the view that overalternations arise because people erroneously believe random processes to be “self-correcting” so as to sustain (accurately perceived) global properties of long-run random sequences, such as equal numbers of heads and tails. One source for such a misconception would be a failure to distinguish between sequences generated with and without replacement (e.g., Ayton et al., 1989; Bar-Hillel & Wagenaar, 1991; Rabin, 2002). In their explicit judgments, the majority of our participants were aware of the fact that heads or tails were equally likely on the next trial. Again, overalternations by those participants, at least, require some other explanation.

In summary, the present data seem difficult to explain on other accounts of the GF and overalternations, though some of them (in particular an erroneous belief in self-correction in random processes) may well play a role in the minority who did endorse the GF, in the same way that they may play a role in understanding the behavior of problem gamblers (e.g., Ladouceur et al., 2001). Even in this case, however, we maintain that a full appreciation of randomness perception will require further study of the links between actual experience and conceptual knowledge of the abstract properties of random processes.

We contend also that such links (and the possible tensions between implicit and explicit measures that it can give rise to) will be important for other human cognitive biases. Rather than interpret such behaviors as fallacies, we suggest that a deeper insight into human cognition can be obtained through the assumption that cognition is rational under constraints, and that people are seeking to make sensible inferences from their experience (Hahn, 2014; Howes, Lewis, & Vera, 2009; Howes, Warren, Farmer, El-Deredy, & Lewis, 2016; Lewis, Howes, & Singh, 2014). The present example of (mis)perceiving randomness in the form of the GF suggests perhaps that there is limited insight to be gained from cold, hard evaluation of human rationality (or lack thereof) against unattainable performance goals. Instead the project of trying to understand human behavior will likely benefit greatly from trying to establish causal links between cognitive constraints and behavior.

## Conclusion

To address the question posed in the title: explicit belief in the GF may be far less widespread than typically assumed. Simple behavioral measures such as ARs are not sufficient to establish bias, and what bias they show seems to be based on people’s actual, limited, experience of random outputs. Rather than interpreting behaviors like the GF as biased or irrational, it may be more productive to explore what they reveal about the constraints that limit otherwise adaptive processes.

## Figures and Tables

**Figure 4 fig4:**
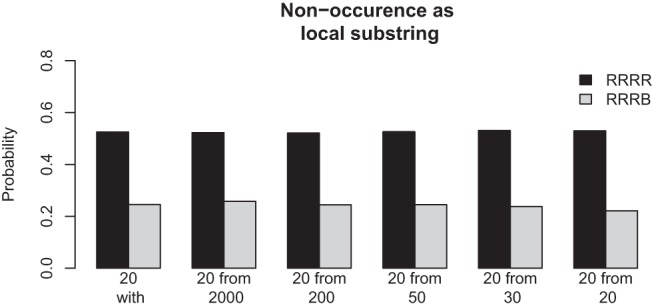
Results of simulations to examine the relative probability that the local substrings of red (R) and blue (B) balls will not be contained within a global sequence of length 20 for the case of sampling with replacement (leftmost bars) and sampling without replacement from urns with decreasing initial numbers of red and blue balls (urn size of initially 2000, 200, 50, 30, and 20, respectively). Figure from Hahn (2014) p. 232.

## Footnotes

1 Note that it is crucial to the model that the sliding window moves incrementally through the sequence (as is implied, e.g., in tests of short term memory such as the N-back test, see, e.g., Kane, Conway, Miura, & Colflesh, 2007). If the attentional/STM window were to “jump” through the global sequences in nonoverlapping, sequential chunks (e.g., HHTHTHTT would be broken up into just HHTH and THTT) then successive local sequences would be entirely independent and the expected values of their occurrence would be equal across all possible sequences just as they are when *k* = *n*.

2 This discussion of differences in wait time and nonoccurrence probability generalizes to other values of *k* and *n* for *k* < *n* < ∞ (see Hahn & Warren, 2009). However, the choice of length 4 subsequences in Figures 1 and 2 is not accidental. It has been suggested that the effective storage capacity limit for short term memory is 4 ± 1 item (when performance strategies such as rehearsal that might differ between participants are prevented; Cowan, 2001, 2010).

3 We report generalized η^2^ as the measure of effect size (Bakeman, 2005; Olejnik & Algina, 2003).

4 Rapoport and Budescu (1997) provide an implementation of Kareev’s (1992) account with the difference that whereas Kareev assumes an unbiased underlying conception of randomness, Rapoport and Budescu’s model implements “representativeness” by modeling STM based output selection through sampling without replacement.

5 The same is true of other performance limitation based accounts such as, for example, Treisman and Faulkner (1987).
